# Well-Plate μFASP for Proteomic Analysis of Single Pancreatic Islets

**DOI:** 10.1021/acs.jproteome.2c00047

**Published:** 2022-03-16

**Authors:** Friederike A. Sandbaumhüter, Mariya Nezhyva, Olle Eriksson, Adam Engberg, Johan Kreuger, Per E. Andrén, Erik T. Jansson

**Affiliations:** † Department of Pharmaceutical Biosciences, Uppsala University, Uppsala 751 24, Sweden; ‡ Department of Medical Cell Biology, Uppsala University, Uppsala 751 23, Sweden; § Science for Life Laboratory, Spatial Mass Spectrometry, Uppsala University, Uppsala 751 24, Sweden

**Keywords:** filter-aided sample preparation, islets of Langerhans, liquid chromatography−mass spectrometry, peptidomics, proteomics

## Abstract

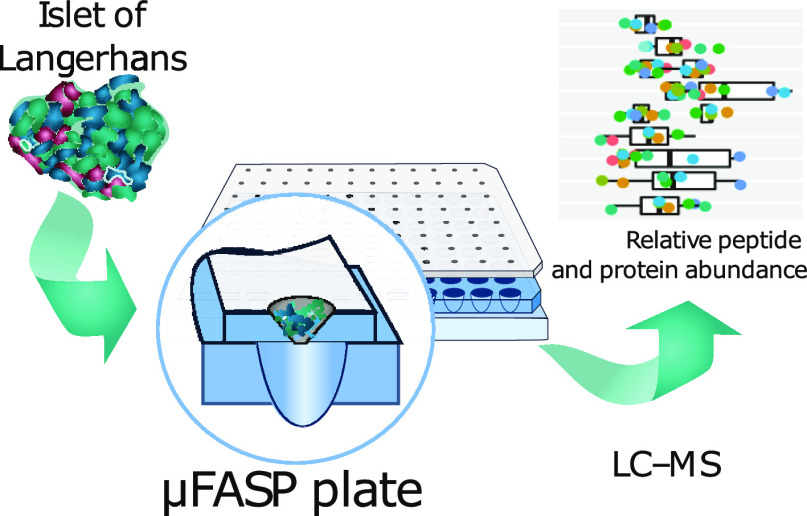

Filter-aided sample preparation (FASP) is widely used in bottom-up proteomics for tryptic digestion. However, the sample recovery yield of this method is limited by the amount of the starting material. While ∼100 ng of digested protein is sufficient for thorough protein identification, proteomic information gets lost with a protein content <10 μg due to incomplete peptide recovery from the filter. We developed and optimized a flexible well-plate μFASP device and protocol that is suitable for an ∼1 μg protein sample. In 1 μg of HeLa digest, we identified 1295 ± 10 proteins with μFASP followed by analysis with liquid chromatography–mass spectrometry. In contrast, only 524 ± 5 proteins were identified with the standard FASP protocol, while 1395 ± 4 proteins were identified in 20 μg after standard FASP as a benchmark. Furthermore, we conducted a combined peptidomic and proteomic study of single pancreatic islets with well-plate μFASP. Here, we separated neuropeptides and digested the remaining on-filter proteins for bottom-up proteomic analysis. Our results indicate inter-islet heterogeneity for the expression of proteins involved in glucose catabolism, pancreatic hormone processing, and secreted peptide hormones. We consider our method to provide a useful tool for proteomic characterization of samples where the biological material is scarce. All proteomic data are available under DOI: 10.6019/PXD029039.

## Introduction

Filter-aided sample preparation (FASP) is an established sample preparation procedure for bottom-up proteomics. Protein reduction, alkylation, and enzymatic digestion are carried out on centrifugal filter devices, while simultaneously salts, lipids, and other contaminants are washed away. In bottom-up proteomics, the resulting peptides are afterward separated by liquid chromatography (LC) or capillary electrophoresis (CE) and analyzed with mass spectrometry (MS). The first FASP protocol was published in 2005 by Manza et al.^1^ and has since then been modified several times.^2,3^

Advantages over in-solution and in-gel digestion are minimized need of sample handling, removal of detergents and other contaminants, and suitability for different digestion conditions.^1−3^ On the other hand, FASP is a time-consuming procedure and because of incomplete peptide recovery, it requires ∼10 μg of the protein starting material.^4,5^ Below that, the reproducibility and the proteome coverage are low. In cases of (sub-) populations of cells, micro-dissected tissues, and fractions of a specific cell-type, the starting material is limited. Consequently, a high number of human and animal donors are required. Furthermore, only an ∼100 ng fraction of the starting material is finally injected into the LC– or CE–MS system. Thus, different variations of the FASP method including additives such as deoxycholic acid, polyethylene glycol, dextran, or polyvinylpyrrolidone as well as chemically passivated filters were introduced in order to improve the proteome coverage for samples with low protein concentrations.^6,7^ Recently, Zhang et al. developed a miniaturized method based on the FASP principle where sample loss is decreased by reducing the filter area.^8^ Alternative approaches for low protein amounts are single-pot-solid-phase-enhanced sample preparation, nanodroplet processing in one pot for trace samples, nanoparticle-aided nanoreactor for nanoproteomics, in-stagetip digestion,^9−13^ and on-microsolid-phase extraction tip-based sample preparation. These methods showed good proteome coverage and quantitative reproducibility with 1–20 μg protein and simultaneously greatly reduced preparation times.^4^

With a ≲0.4 μg protein content, individual pancreatic islets of Langerhans are below the appropriate limit for standard FASP.^14^ These micro-organs consist of different endocrine cells that produce, store, and secrete pancreatic hormones to regulate glucose homeostasis. The main secreted hormones are insulin and glucagon released from β-cells and α-cells, respectively. Further, loss of β-cell mass and function may lead to the chronic metabolic disease diabetes. Interindividual differences in composition and function of islets of Langerhans have been hypothesized to play an important role in the maintenance of glucose homeostasis.^15^ However, single islet analysis is required to delineate individual islets and understand the functional consequences of islet heterogeneity.^14−18^

Here, we provide a miniaturized FASP method for bottom-up proteomic analysis of individual islets of Langerhans. The goal of this work was (i) to design and optimize a flexible well-plate μFASP device that is compatible with basic laboratory equipment and does not require long preparation times, (ii) to develop a protocol that increases the proteome recovery for samples with a limited starting material while simultaneously reducing the sample preparation time, and (iii) to apply it to the peptidomic and proteomic characterization of single islets of Langerhans.

## Experimental Section

### Chemicals and Reagents

Dithiothreitol (DTT), iodoacetamide (IAA), and HEPES were purchased from Sigma-Aldrich Chemie, Steinheim, Germany, and urea and NH_4_HCO_3_ from Acros Organics, Geel, Belgium. Trypsin, protease inhibitor, dimethyl sulfoxide, Dulbecco’s Modified Eagle Medium, fetal bovine serum albumin, and the Coomassie Plus (Bradford) Assay Kit were from Thermo Scientific, Waltham, MA. Tris(hydroxymethyl)aminomethane hydrochloride (Tris), LC–MS grade water, acetonitrile, and formic acid were obtained from Fisher Scientific, Geel, Belgium. RapiGest SF Surfactant was purchased from Waters Corporation, Milford, MA.

### Design of the μFASP Plate

**Figure 1:**
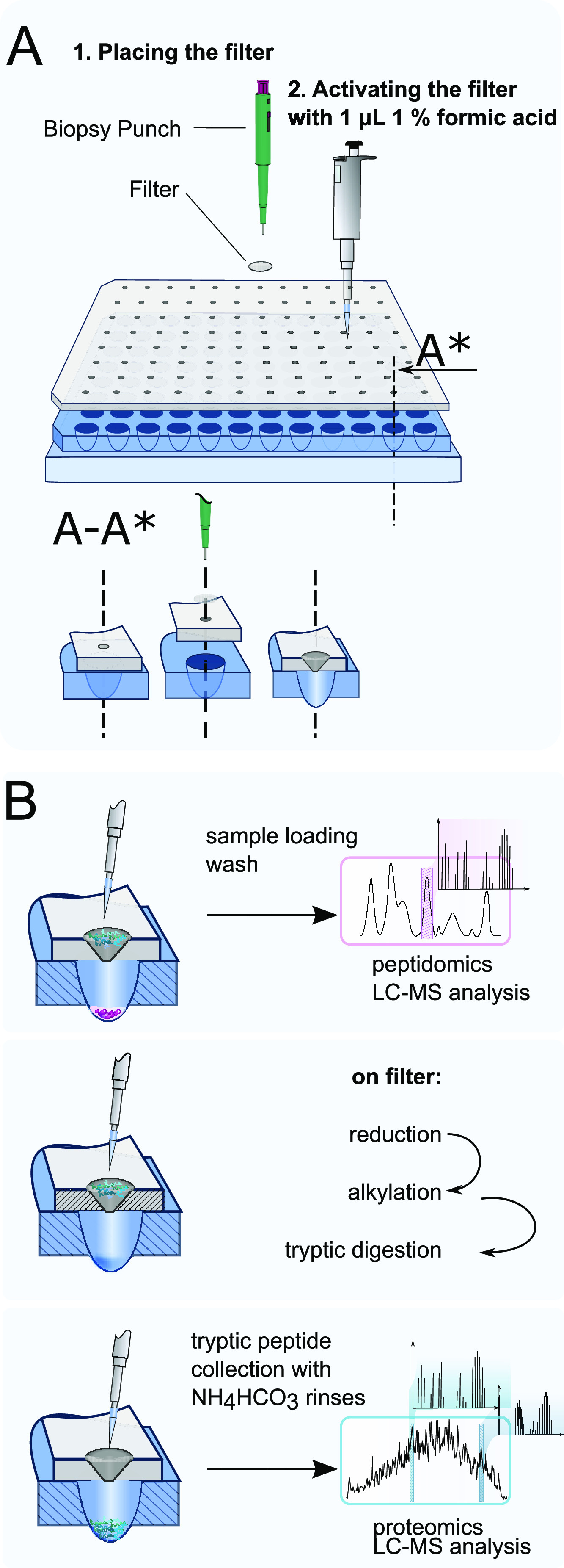
(A) Design and assembly of the well-plate μFASP. The filters are placed with the help of a biopsy punch and afterward activated with formic acid. (B) Overview of the workflow. Sample loading is followed by washing, reduction, and alkylation. After tryptic digestion, the resulting peptides are collected in a 96-well plate. Tryptic peptides as well as the flow-through from the sample loading step can be analyzed with LC–MS.

The μFASP plate was designed to be compatible with the 96-well plate format and hold ⌀1 mm filters. The μFASP plate was drawn in Fusion 360 (Autodesk Inc, CA) and machined from polycarbonate using a Carbide Nomad 883 Pro (Carbide 3D, IL) computer numerical-controlled (CNC) mill. Toolpaths for the CNC mill were created using Fusion 360, and Carbide Motion (Carbide 3D, IL) was used as the machine control software. The μFASP plate has 96 mounting positions for filters of ⌀ 1 mm. The filters can be punched out and mounted in the μFASP plate by using a 1 mm Miltex biopsy punch (Ted Pella Inc, CA). The dimensions of the filter mounting positions match the measures of the biopsy punch and the plunger. The filter mounting position for each filter in the μFASP plate has a shallow counterbore that aligns the biopsy punch with a deeper counterbore that serves as a press fit for the filters. Filters were mounted in the plate by aligning a biopsy punch containing a filter to the shallow counterbore and pressing the plunger of the biopsy punch to push the filter in to a press fit in the deeper counterbore (Figure 1A, S1A–D).

### Lysis of HeLa Cells and Determination of Total Protein Content

HeLa cells cultured in Dulbecco’s modified Eagle medium complemented with 10% fetal bovine serum and 2 mM glucose were harvested using accutase, washed with PBS 3 times, and afterward lysed using tip sonication at 4 °C (pulse 10 × 1 s, rest 1 s, amplitude 30%; Vibra cell ultrasonic processor with a 3 mm probe; Sonics, Newton, CT) in a buffer containing 20 mM HEPES, 6 M urea, 1 μg/mL RapiGest SF Surfactant, and 1% (v/v) protease inhibitor.

The total amount of protein in the lysate was measured with the Coomassie Plus (Bradford) Assay Kit according to the manufacturer’s instructions. Briefly, 10 μL of each calibration standard (25 to 2000 μg/mL bovine serum albumin in lysis buffer), lysis buffer (blank), and the sample were mixed with 300 μL of Coomassie Plus reagent and then incubated at room temperature for 10 min. The absorbance was measured at 595 nm. The blank corrected values for the calibration were plotted against the concentrations, and a standard curve was fitted by linear regression that was further used for calculating the sample concentrations.

### Characterization of the μFASP Plate

HeLa lysate was mixed with a buffer (pH 8.5) consisting of 8 M urea and 100 mM Tris (1:1). 8 mM DTT was added followed by an incubation at 56 °C for 15 min. After adding 50 mM IAA and incubation at room temperature for 20 min, a 16 h incubation at 37 °C was started by adding trypsin (enzyme–protein ratio 1:50 [w/w]). Aliquots of the digest corresponding to 1 μg of protein were loaded onto 96 μFASP filters that were inserted into the μFASP plate and activated with 1 μL of 1% formic acid. Upon centrifugation (500 g, 5 min), two washes with 1 μL 50 mM NH_4_HCO_3_ followed. 100 μL of the Coomassie (Bradford) reagent was added to the eluate and the absorbance was measured after 10 min of incubation at room temperature. Aliquots containing 20 μg of proteins were loaded on regular FASP filters and eluted according to the FASP protocol. A fraction of the eluate was transferred on a 96 well-plate and analyzed with the Bradford assay. A calibration was done with bovine serum albumin in a range of 0.5–10 μg/mL.

### Sample Preparation with FASP

For protein purification and tryptic digestion, different amounts of total protein (20, 10, 5, 1, and 0.5 μg) were transferred onto centrifugal filter units (Microcon-30 kDa; Merck, Darmstadt, Germany) and processed as reported previously.^19^ Briefly, washing with a buffer containing 8 M urea and 100 mM Tris (pH 8.5) was followed by the reduction with 8 mM DTT (incubation at 56 °C for 15 min). For alkylation, an incubation with 50 mM IAA at room temperature was performed for 20 min. Excess IAA was removed with 8 mM DTT (incubation at 56 °C for 15 min). After each incubation, the samples were washed with the urea and Tris containing buffer twice (centrifugation at 14,000*g* for 15 min). After washing the filter with NH_4_HCO_3_ 3 times, trypsin was added [enzyme–protein-ratio 1:50 (w/w)] and the samples were incubated in a wet chamber at 37 °C for 16 h. Resulting peptides were washed from the filter by adding 50 mM NH_4_HCO_3_ twice, each time followed by centrifugation at 14,000*g* for 10 min. Before drying the samples at 45 °C, trifluoroacetic acid was added to a final concentration of 1% (v/v). Afterward, the samples were reconstituted in 3% acetonitrile and 0.1% formic acid in water to a final concentration of 150 ng protein/μL.

### Sample Preparation with Well-Plate μFASP

Filter membranes (molecular cut-off, 30 kDa, Hydrosart, Sartorius Stedim Biotech GmbH, Göttingen, Germany) were inserted into the μFASP plate by using a biopsy punch. The plate with mounted filters was installed on a 96-well plate, immediately wetted with 1 μL 1% formic acid, and centrifugated at 500*g* for 3 min (Figures 1A, S1D). HeLa cell lysates with different amounts of protein (5, 2.5, 1, 0.5, and 0.25 μg) were loaded onto the filters followed by another centrifugation (500*g*, 3 min) of the whole well-plate μFASP device. All loaded samples were processed in parallel. The volumes pipetted onto the filters during the following steps were always 1 μL. Each step was followed by centrifugation at 500*g* for 3 min. After washing the filters with a buffer containing 8 M urea and 100 mM Tris (pH 8.5), DTT (8 mM) was added and the whole device incubated at 37 °C for 15 min. For alkylation, the samples were incubated with 50 mM IAA at room temperature for 20 min and then again with 8 mM DTT at 37 °C for 15 min. All incubation steps were followed by washing the filters twice with the urea-Tris buffer. Before adding trypsin [enzyme–protein ratio 1:50 (w/w)], the filters were washed with 50 mM NH_4_HCO_3_ 3 times, and the μFASP plate was placed on top of a new 96-well plate. After 16 h of incubation at 37 °C in a wet chamber and immediate centrifugation at 500*g* for 3 min, the filters were washed with 50 mM NH_4_HCO_3_ twice and the tryptic peptides were collected in the 96-well plate by centrifugation at 500*g* for 5 min each. A solution of 3% acetonitrile and 0.1% formic acid in water was added to a final protein concentration of 150 ng/μL (Figure 1B).

### Well-Plate μFASP Preparation of Single Islets of Langerhans

All animal experiments were approved by the regional animal ethics committee in Uppsala, Sweden (ethics approval number 5.9.18-03603/2018). Single islets of Langerhans from mice were separated in 2 μL of medium into PCR tubes. 2 μL of the lysis buffer was added and the samples were lyzed by freezing–thawing them twice. The lysates were transferred in two portions onto the activated μFASP plate. The flow-through from the loading and the first washing step with urea buffer was collected and injected directly into the LC–MS system. Then, it proceeded according to the protocol described above (Figure 1B).

For in-solution digestion, the same reagents as described before were used. First, 1 μL of DTT was added followed by an incubation at 37 °C for 15 min and then 1 μL of IAA. After 20 min of incubating at room temperature, trypsin was added [enzyme–protein ratio 1:50 (w/w)]. The incubation at 37 °C overnight was stopped by adding 50 mM NH_4_HCO_3_ and trifluoroacetic acid [final concentration of 1% (v/v)]. Afterward, the samples were dried and reconstituted in 2.7 μL of a solution containing 3% acetonitrile and 0.1% formic acid in water.

### UPLC-MS/MS Analysis

For tryptic peptide analysis, a nanoAcquity UPLC system equipped with a C18, 5 μm, 180 μm × 20 mm trap column and a HSS-T3 C18 1.8 μm, 75 μm × 250 mm analytical column (Waters Corporation, Manchester, UK) was coupled to a Synapt G2 Si HDMS mass spectrometer with an electrospray ionization source (Waters Corporation, Manchester, UK). Mobile phase A contained 0.1% formic acid and 3% dimethyl sulfoxide in water and mobile phase B 0.1% formic acid and 3% dimethyl sulfoxide in acetonitrile. 300 ng of protein was injected in trapping mode. The peptides were separated at 40 °C with a gradient run from 3 to 40% (v/v) mobile phase B at a flow rate of 0.3 μL/min over 120 min. Via the reference channel, a lock mass solution composed of [Glu1]-fibrinopeptide B (0.1 μM) and leu-enkephalin (1 μM) was introduced every 60 s. Peptide analysis was performed in positive ionization mode using the ultra-definition MSE (UDMSE) approach.^19,20^ The reproducibility and stability of the method were controlled with a commercially available HeLa digest (Thermo Scientific, Waltham, MA).

### Data Processing and Statistical Analysis

ProteinLynx Global Server (PLGS) (version 3.0.3, Waters Corporation, Milford, MA) was used for data processing. The HeLa samples were searched with a false discovery rate (FDR) of 0.01 against a randomized UniProt human database (Uni-ProtKB version 14/01/2020) with carbamidomethyl cysteine set as a fixed modification; acetyl lysine, C-terminal amidation, asparagine deamidation, glutamine deamidation, and methionine oxidation as variable modification; and trypsin as the digest reagent. One missed cleavage was allowed. Minimum peptide matches per protein were 2, and minimum ion matches per peptide and protein were 1 and 3, respectively. The islet digests were searched with the same parameter but without any modification settings against a randomized mouse database (UniProtKB version 14/01/2020). Both, tryptic digests of islets and collected flow through samples, were additionally searched against the SwePEP database.^21^

Identified proteins inferred from bottom-up proteomic analysis using PLGS were quantitated with the TOP3 method obtained directly from ISOQuant 1.83 as described elsewhere.^19,20^ ISOQuant settings are provided in Supporting Information Table S1.

Identified peptide hormone products from intact peptidomic analysis were quantitated with the iBAQ method.^22^ Briefly, intensities for all peptide products quantitated with ISOQuant and assigned to a preprohormone in the SwePEP database were imported to R. The data were filtered where only quantitated peptide sequences matching with prohormone processing annotated in UniProt were retained. The peptide product intensities were then obtained as the mean of all intensities for the retained and matching peptides per peptide product.

Statistical analysis was done with Student’s *t*-test. A *p*-value < 0.05 was considered as statistically significant.

## Results and Discussion

### Development of the Well-Plate μFASP

The here-presented well-plate μFASP was developed and optimized for samples containing 1 μg of protein. Zhang et al. showed that the loss of proteomic information for low amounts of the protein starting material decreases along with a reduced filter area.^8^ Based on that, the filter area of 118.823 mm^2^ of the centrifugal filter units used for FASP was reduced to 0.785 mm^2^. The design of the μFASP plate combined with the use of the biopsy punch ensures a correct, reproducible, and easy placement of the filters in a single step and their accessibility for all necessary washing and reaction solutions. Mounting one filter takes approximately 3 s and does not require a lot of training. In comparison, the assembling of the microreactors by Zhang et al. is a multistep procedure that is time- and training-intense.^8^ This includes also the risk of reproducibility issues and consequently differences in the performance.

Furthermore, the design offers a high degree of flexibility. Filters of different pore sizes and/or material can be used in parallel or changed quickly. In the here-presented experiments, Hydrosart filter membranes with a molecular cut-off of 30 kD were used. This hydrophilic filter material prevents losses through protein binding to the filter and is therefore a suitable material for general proteomic applications. The ratio between performance and centrifugation time was previously shown to be best for a molecular cut-off of 30 kD.^8,23^

The installation on a 96-well plate lays the foundation for high-throughput applications and automatic sample injection directly from the collecting plate. It was tested to be compatible with a multichannel pipette. In an experiment where the HeLa digest was loaded onto all 96 μFASP filters, the analysis of the eluate with the Bradford assay showed the reproducibility of the performance. The mean of the absorption was 0.217 ± 0.038 and the relative standard deviation (RSD) 14% (Supporting Information Table S6). No differences between the samples eluted from the corner positions of the μFASP well plate and the other positions were found. The corner positions serve also as locating features and have therefore slightly different dimensions. 61% of the starting material was found in the case of μFASP and 63% in the case of standard FASP.

We used conical-shaped well plates that facilitate the collection of the digest. Polycarbonate as a plate material is chemical-resistant and allows to reuse the μFASP plate many times. Other FASP-based methods that were developed for automated high-throughput analysis involve commercially available filter well-plates.^24^ They are limited in the choice of filter types, and costs are higher than for the reusable μFASP plate. Further advantages are the low consumption of reagents as only 1 μL of washing and reaction solutions per step are required and a shorter preparation time. With centrifugation times of 3–5 min per step, the total time for centrifugation is 55 min compared to 215 min necessary for the FASP protocol.

Loading HeLa lysates corresponding to less than 5 μg of protein onto a conventional FASP device resulted in a significantly decreased number of identified proteins compared to higher protein amounts (Table 1, Supporting Information Table S2). In samples with 20, 10, and 5 μg of protein as the starting material, 1395 ± 4, 1392 ± 2, and 1323 ± 10 proteins and 9000 ± 78, 8381 ± 48, and 7687 ± 206 peptide-spectrum matches (PSMs) were identified. In contrast, with 1 and 0.5 μg, only 524 ± 5 and 316 ± 1 proteins and 2864 ± 14 and 1620 ± 26 PSMs were found. Due to non-specific binding to the filter and irreversible protein aggregation, about 50% of the starting material gets lost during the sample preparation to mainly affect the analyses of samples with low protein contents (Table 1).^4−6^

**Table 1:** Identified Proteins and Peptides (Mean ± SD) after Using the Conventional FASP Protocol (*n* = 3) or the μFASP Plate (*n* = 5) for Preparation of Different Amounts of Protein

sample/μg	method	protein ID^a^	PSMs^b^	C2^c^/min	C3^d^/min
20	FASP	1395 (±4)	9001 (±78)	10	10
10	FASP	1392 (±2)	8381 (±48)		
5	FASP	1323 (±10)	7687 (±206)		
1	FASP	524 (±5)	2864 (±14)		
0.5	FASP	316 (±1)	1620 (±26)		
1	μFASP	423 (±29)	1794 (±328)	3	3
1	μFASP	1144 (±11)	6793 (±417)	3	5
5	μFASP	68 (±6)	158 (±32)	5	5
2.5	μFASP	140 (±41)	519 (±234)		
1	μFASP	1295 (±10)	7940 (±447)		
0.5	μFASP	659 (±15)	3354 (±350)		
0.25	μFASP	947(±28)	5255 (±865)		

a Protein identifications.

b Peptide spectrum matches.

c Second centrifugation step.

d Third centrifugation step.

### Optimization of the Well-Plate μFASP Protocol

**Figure 2:**
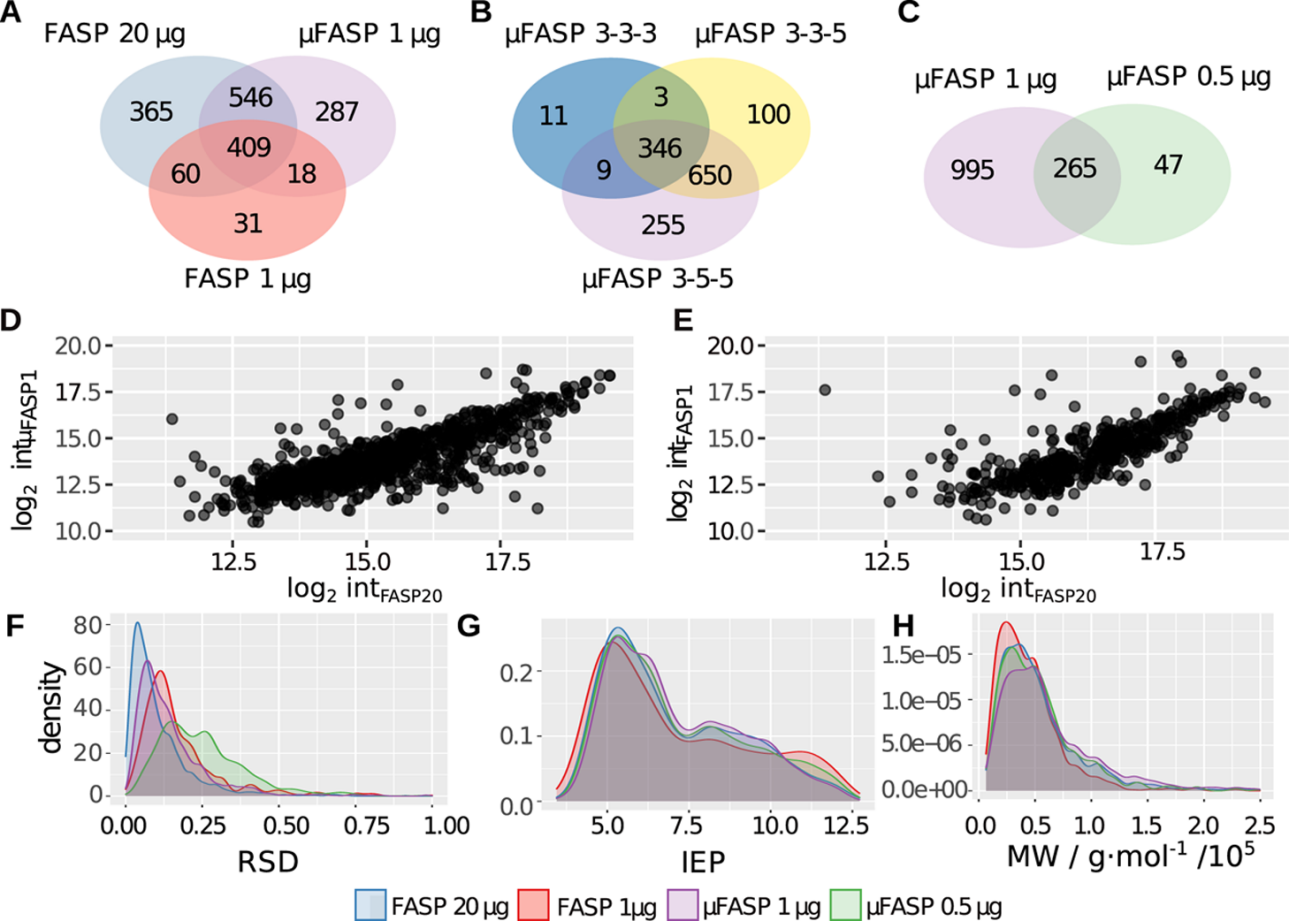
Venn diagrams of (A) number of identified proteins after FASP for 20 and 1 μg of starting material and after μFASP for 1 μg of starting material; (B) number of identified proteins for 1 μg of starting material prepared with μFASP using centrifugation times of 3, 3, and 3 min, 3, 3, and 5 min, and 3, 5, and 5 min for sample collection; (C) number of identified proteins for 1 and 0.5 μg of starting material prepared with μFASP. Correlation plots for 20 μg FASP vs 1 μg after (D) μFASP and (E) FASP, respectively. (F) RSD values for protein abundances were calculated for 1 and 20 μg of starting material prepared with FASP and 1 and 0.5 μg of starting material prepared with μFASP. For the same preparations (G), the isoelectric point (IEP) and (H) the molecular weight (MW) were plotted against density estimations.

The protocol for the well-plate μFASP is adapted from the standard FASP protocol that was previously used to study the effect of a brain-targeting somatostatin peptide on different brain regions.^19^ Based on previous findings by Distler et al., reduction and alkylation in the protocol are performed directly on the filter.^3^ Further, a second incubation with DTT ensures the removal of IAA. Thus, it is important that the reduction and alkylation reagents can reach the filter membrane. In accordance with the FASP protocol, several washing steps with a buffer containing Tris and urea were performed in order to remove contaminants and remaining DTT and IAA. In total, three centrifugation steps are used for collecting the peptides after enzymatic digestion on the μFASP filter, one directly after incubation and one after each of the two washing steps with ammonium bicarbonate. Spinning for 3 min each (μFASP 3-3-3) resulted not only in volumes much smaller than the 3 μL that was loaded during the sample collection process onto the filter but also in a low number of identification and low reproducibility (Table 1). By first increasing the third centrifugation step to 5 min (μFASP 3-3-5) and then also the second centrifugation step (μFASP 3-5-5), we obtained a higher number of identifications and a lower variability between the replicates (Table 1, Figure 2A and Supporting Information Table S2). When also the first centrifugation step was set to 5 min, some filters were removed from their places during centrifugation. The dryness of the filters after overnight incubation at 37 °C can be a reason for their dislocation.

In contrast, before the following centrifugation steps, the filters were wetted with ammonium bicarbonate solution. Thus, this setting was not used further and μFASP 3-5-5 in the following simply named as μFASP was implemented in the final protocol. 1395 proteins could be detected in 20 μg by using the conventional FASP protocol. 955 of these were also detected in 1 μg with the final μFASP method and only 469 after preparing 1 μg on an FASP filter. 365, 287, and 31 proteins were exclusively detected in 20 μg after FASP, in 1 μg after μFASP, and in 1 μg after FASP, respectively (Figure 2B). Thus, FASP and μFASP provide for their optimal loading amounts of 20 and 1 μg protein, respectively, comparable results. The same agreement between the standard and the miniaturized methods was also found for the FASP microreactors.^8^ The absolute numbers of the two miniaturized methods are not comparable because of different protocols and instrumentations. Increasing (2.5 and 5 μg) as well as decreasing (0.5 μg) the amount of loaded protein on the μFASP plate led to a decreased number of identified proteins (Table 1). Overloading and blocking the filter on one hand and a low peptide recovery comparable to low protein amounts on a regular FASP filter on the other hand are probable. For the regular FASP device, it was shown that loading more than 100 μg of protein results in lower numbers of identified proteins and peptide spectrum matches.^25^ Following the linear relationship between filter area and loading limit, an estimation for the well-plate μFASP can be made. On the other hand, centrifugation times and speed, number of washing steps, and type and volume of washing reagents affect the final yield as well.^25^ With 0.5 μg of starting material, however, 659 ± 15 proteins, respectively, were detected, and with that, significantly more than with 0.5 μg protein on a regular FASP filter (316 ± 1) (Table 1). Comparing the results for 1 and 0.5 μg of protein prepared with the μFASP protocol showed that most of the proteins that were found in the 0.5 μg sample were also detected in the 1 μg sample (Figure 2C). The correlation between the mean intensities of 20 μg after FASP and 1 μg after μFASP can be described with a correlation coefficient of 0.764 (Figure 2D). For 1 μg after FASP, it is only 0.592, and the intercept is shifted to higher values, indicating a slight underestimation of intensities compared to what 20 μg of the starting material yields (Figure 2E). In order to see how the system behaves at the limit, e.g., in terms of pipetted and collected volumes, 0.25 μg of protein was loaded onto the filter. As this was below the standard LC injection amount of 300 ng, the whole eluate was injected onto the column. Interestingly, the number of detected proteins was higher than the one for 0.5 μg of the starting material. Reasons are multifactorial, and it can only be hypothesized that basic processes on the filter changed with the low volume and protein amount. More importantly, at all concentration levels, the workflow showed good reproducibility.

While the RSD values for protein intensities for 20 μg with FASP and 1 μg with μFASP are mostly overlapping, the maximum of the distribution for 1 μg with FASP is clearly shifted toward higher RSD values (Figure 2F). Furthermore, Figure S2 shows good correlations between the replicates of the 20 μg FASP samples (correlation coefficients 0.975–0.988) and 1 μg μFASP samples (0.943–0.992). The correlation plots between the replicates of the two different preparation methods (0.735–0.782) show for all runs a slight trend of underestimating proteins from the center of the intensity range in the μFASP; the residual standard error of the linearized model of the data shows that the deviation of log_2_(int) is ∼1.

Comparing the physicochemical properties of the detected proteins for FASP preparations of 20 and 1 μg and μFASP preparations of 1 and 0.5 μg confirms biases toward lower and higher isoelectric points (IEPs) as well as lower MWs for 1 μg compared to 20 μg after FASP that were reported by Sielaff et al.^4^ (Figure 2G,H). Interestingly, 1 μg after μFASP follows the behavior of 1 μg after FASP for high IEPs and MWs. For low IEPs, it is between 1 and 20 μg after FASP, whereas the results for 0.5 μg after μFASP are similar to those of 20 μg for high IEPs and to 1 μg after FASP for low IEPs.

### Proteomic Characterization of Single Islets of Langerhans by Using the Well-Plate μFASP

**Figure 3:**
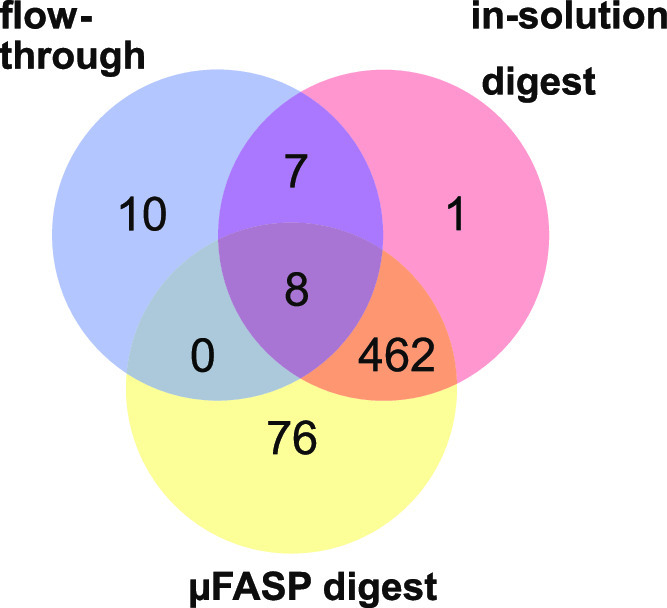
Venn diagram comparing detected proteins in single islets after in-solution digestion, μFASP, and flow-through analysis.

**Figure 4:**
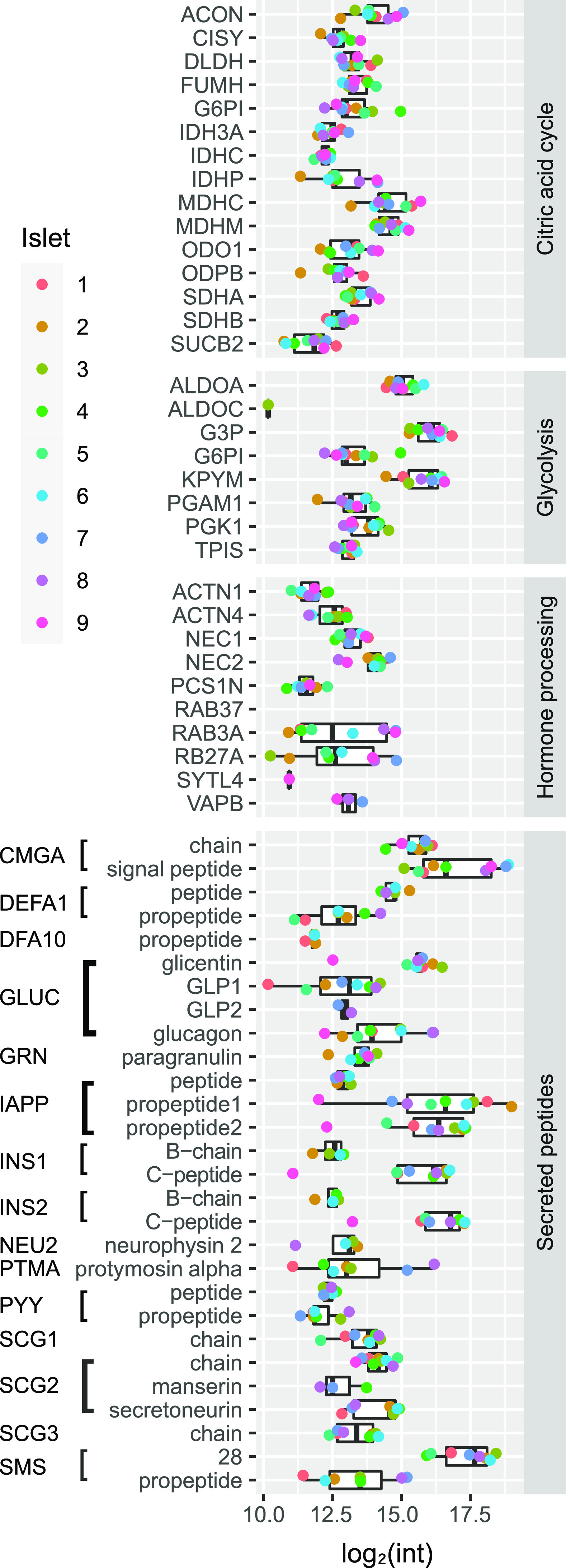
Islet heterogeneity shown by expression levels of proteins involved in the citric acid cycle, glycolysis, and hormone processing as well as secreted peptides in nine individual islets.

Islets of Langerhans are heterogeneous in size, architecture, cellular composition, and glucose sensitivity.^16,17^ Often for proteomic analysis, several islets are pooled together because one islet typically contains ≲0.4 μg of protein.^14^ Since islet heterogeneity is suspected to play a role in the development of metabolic diseases such as diabetes, analysis of the ensemble average obtained from pooled samples may mask important information.^14,18,26^ A previously published deep proteome analysis of single islets was done with time-intense replay-LC–MS and showed their qualitative differences.^14^ The here-presented study provides also a quantitative comparison of proteins and peptides detected in single islets. Single islets of Langerhans were prepared and digested with the well-plate μFASP and, in turn, compared to islets that were prepared according to a standard in-solution digestion protocol (Supporting Information Table S3). 478 proteins were identified with the in-solution digestion including 15 highly abundant pancreatic hormones (Figures 3, 4). With μFASP, it was expected that these hormones pass the molecular cut-off filter after sample loading due to their small MW. Removing highly abundant pancreatic hormones, insulin in particular, from the rest of the sample increases the chance to detect lower abundant proteins since this increases the molar fraction of each protein retained in the sample. This is important for a complete characterization of healthy and (pre-)diabetic islets of Langerhans and the identification of pathways that are affected in the early stages of diabetes. Islet amyloid peptide, glucagon, secretogranin 2 and 3, chromogranin A, prothymosin α, and peptide YY, however, were detected in the μFASP digest (Figure 3). The flow-through collected after sample loading was analyzed using the same LC–MS and processing methods as for the two types of islet digests, and 25 neuropeptides were detected (Supporting Information Table S4). Combining the results from the μFASP digest analysis and the flow-through analysis led, in total, to the identification of 76 proteins and 10 neuropeptides more than with the in-solution digestion while no information got lost (Figure 3). This demonstrates that the well-plate μFASP approach shows a better performance than in-solution digestion even with protein amounts that are below its optimal loading amount of 1 μg. Among the detected proteins are proteins involved in glucose uptake, glucose catabolism, and insulin exocytosis. Figure 4 shows proteins from glycolysis, citric acid cycle, and pancreatic hormone processing as well as the secreted pancreatic peptides. Islet heterogeneity is not only visible in the different expression levels of those proteins in the nine individual islets but also in the range of variance for the individual proteins. For pancreatic peptide hormones, the range of the expression levels was analyzed on the individual peptide level with the iBAQ-method.^22^ These peptides differ also in their coverage (Supporting Information Table S5). Comparing their expression levels between the nine single islets shows the range of the interindividual variability and, in turn, highlighting the importance of single islet analysis (Figure 4).

## Conclusions

With the novel well-plate μFASP, we present a flexible tool that can be used to prepare samples where biological material is limited and the amounts of total protein are low, with good reproducibility and proteome coverage for MS-based bottom-up proteomic analysis. Not only the sample preparation time but also the consumption of both reagents and the starting material was reduced when compared to standard FASP. Our peptidomic and proteomic studies of single islets of Langerhans show how this method opens new analytical insights by separating small amounts of the protein sample with a filter into fractions that can be individually analyzed.

## Supporting Information Available

Design of the μFASP plate; correlation plots for the single replicates of FASP and μFASP; ISOQuant parameter; sequence coverage of pancreatic peptides; and Bradford assay absorbance data (PDF)
List of all quantified proteins across all HeLa digests prepared with FASP and μFASP (XLSX)
List of all quantified proteins in μFASP and in solution digests of single pancreatic islets μFASP (XLSX)
List of all peptide intensities detected in the flow-throughs of single pancreatic islets (XLSX)

## Notes

The mass spectrometry proteomics data have been deposited to the ProteomeXchange Consortium via the PRIDE partner repository, accessible through DOI: 10.6019/PXD029039. The authors declare that the Table of Contents graphic is in compliance with the journal’s guidelines.
