# Supplementary material for: Well-Plate μFASP for Proteomic Analysis of Single Pancreatic Islets
Source: J Proteome Res. 2022 Mar 16;21(4):1167–74. doi: 10.1021/acs.jproteome.2c00047 (PMC8981318; doi:10.1021/acs.jproteome.2c00047)
Supplement: Supplementary file 1 — pr2c00047_si_001.pdf [file pr2c00047_si_001.pdf]

Supporting Information

Well-plate μFASP for proteomic analysis of single pancreatic islets

Friederike A. Sandbaumhüter, Mariya Nezhyva, Olle Eriksson, Adam Engberg, Johan Kreuger, Per E. Andrén, Erik T. Jansson

Table of content

Figure S1.	Design of the μFASP plate
Figure S2.	Correlation plots of the single replicates of FASP preparations with 20 μg protein and μFASP preparations with 1 μg protein
Supplementary Table S1.	ISOQuant parameters
Supplementary Table S2.	List of all quantified proteins across all HeLa digests prepared with FASP and μFASP (provided as excel file)
Supplementary Table S3.	List of all quantified protein in μFASP and in solution digests of single pancreatic islets (provided as excel file)
Supplementary Table S4.	List of all peptide intensities detected in the flow throughs of single pancreatic islets (provided as excel file)
Supplementary Table S5.	Sequence coverage of pancreatic peptides
Supplementary Table S6.	Bradford assay absorbance data.

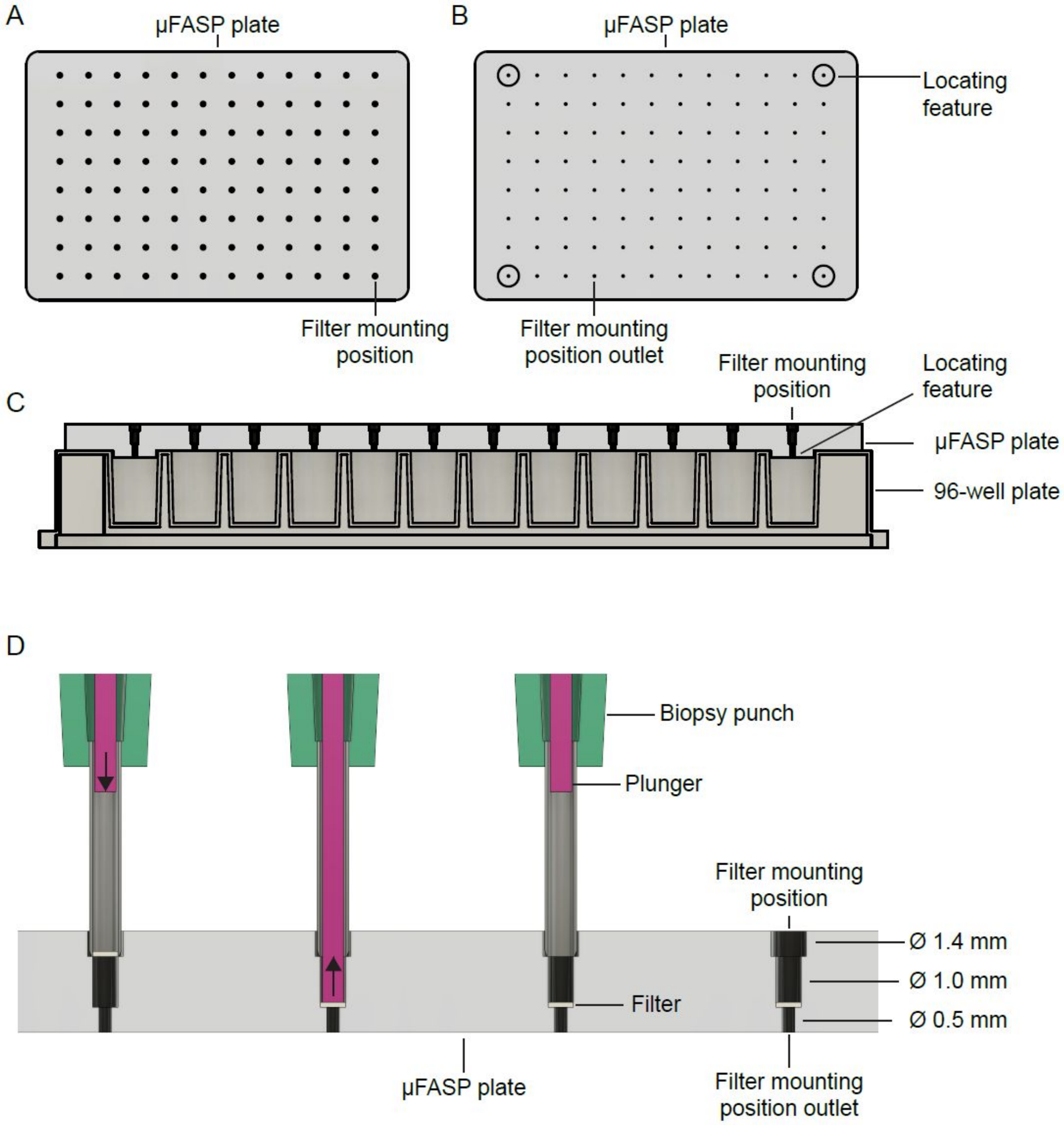

Figure S1. Overview of the µFASP plate. (A) Top view of the µFASP plate showing 96 filter mounting positions. (B) Bottom view of the µFASP-plate showing the outlets of the filter mounting positions and the locating features present on the four outer filter mounting positions. (C) Cross-sectional cutaway view of the µFASP plate assembled on top of a 96-well plate. Locating features of the µFASP plate align it to the 96-well plate. (D) Enlarged view of cross-sectional cutaway view illustrating the stages of mounting filters in the µFASP plate. From left to right, a biopsy punch with a filter is aligned by fitting it into the shallow counterbore. Pressing the plunger down the filter is pushed down into a press fit in the

deeper counterbore. When filter is mounted in deeper counterbore the plunger can be withdrawn. The biopsy punch is removed and the filter has been mounted.

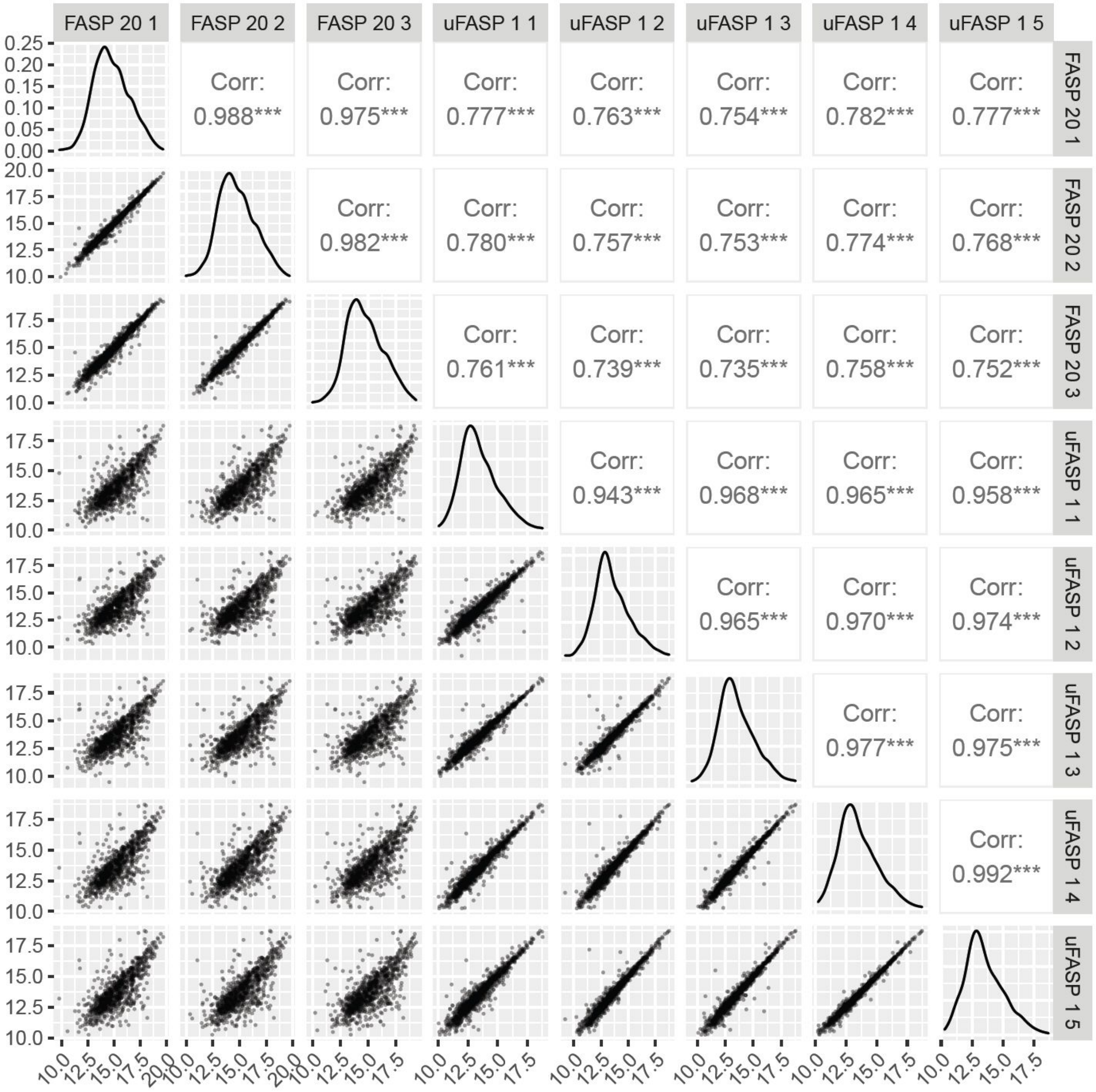

Figure S2. Correlation plots comparing the single replicates for the HeLa digest analysis after FASP with 20 μg protein (n = 3) and μFASP with 1 μg protein (n = 5).

Supplementary Table S1: ISOQuant 1.8 software settings used for label-free quantification analysis of LC–MS data

**parameter**	**value**
isoquant.pluginQueue.name	design project and run ISOQuant analysis
process.peptide.deplete.PEP_FRAG_2	false
process.peptide.deplete.CURATED_0	false
process.peptide.statistics.doSequenceSearch	false
process.emrt.minIntensity	1000
process.emrt.minMass	500
process.emrt.rt.alignment.match.maxDeltaMass.ppm	10
process.emrt.rt.alignment.match.maxDeltaDriftTime	2
process.emrt.rt.alignment.normalizeReferenceTime	false
process.emrt.rt.alignment.maxProcesses	24
process.emrt.rt.alignment.referenceRun.selectionMethod	AUTO
process.emrt.clustering.preclustering.orderSequence	MTMTMT
process.emrt.clustering.preclustering.maxDistance.mass.ppm	6.06E-6
process.emrt.clustering.preclustering.maxDistance.time.min	0.202
process.emrt.clustering.preclustering.maxDistance.drift	2.02
process.emrt.clustering.distance.unit.mass.ppm	6.0E-6
process.emrt.clustering.distance.unit.time.min	0.2
process.emrt.clustering.distance.unit.drift.bin	2
process.emrt.clustering.dbscan.minNeighborCount	2
process.identification.peptide.minReplicationRate	2
process.identification.peptide.minScore	0
process.identification.peptide.minOverallMaxScore	0
process.identification.peptide.minSequenceLength	6
process.identification.peptide.acceptType.PEP_FRAG_1	true
process.identification.peptide.acceptType.IN_SOURCE	false
process.identification.peptide.acceptType.MISSING_CLEAVAGE	false
process.identification.peptide.acceptType.NEUTRAL_LOSS_H20	false
process.identification.peptide.acceptType.NEUTRAL_LOSS_NH3	false
process.identification.peptide.acceptType.PEP_FRAG_2	false
process.identification.peptide.acceptType.DDA	true
process.identification.peptide.acceptType.VAR_MOD	false
process.identification.peptide.acceptType.PTM	false
process.annotation.peptide.maxSequencesPerEMRTCluster	1
process.annotation.protein.resolveHomology	true
process.annotation.peptide.maxFDR	0.01
process.annotation.useSharedPeptides	all
process.normalization.lowess.bandwidth	0.3
process.normalization.orderSequence	XPIR

process.normalization.minIntensity	3000
process.quantification.peptide.minMaxScorePerCluster	0
process.quantification.peptide.acceptType.IN_SOURCE	false
process.quantification.peptide.acceptType.MISSING_CLEAVAGE	false
process.quantification.peptide.acceptType.NEUTRAL_LOSS_H20	false
process.quantification.peptide.acceptType.NEUTRAL_LOSS_NH3	false
process.quantification.peptide.acceptType.PEP_FRAG_1	true
process.quantification.peptide.acceptType.PEP_FRAG_2	false
process.quantification.peptide.acceptType.VAR_MOD	false
process.quantification.peptide.acceptType.PTM	false
process.quantification.peptide.acceptType.DDA	true
process.quantification.topx.degree	3
process.quantification.topx.allowDifferentPeptides	true
process.quantification.minPeptidesPerProtein	1
process.quantification.topx.allowDifferentPeptides	true
process.quantification.maxProteinFDR	0.01

Supplementary Table S2. List of all quantified proteins across all HeLa digests prepared with FASP and μFASP (provided as excel file)

Supplementary Table S3. List of all quantified protein in µFASP and in solution digests of single pancreatic islets (provided as excel file)

Supplementary Table S4. List of all peptide intensities detected in the flow throughs of single pancreatic islets (provided as excel file)

Supplementary Table S5. Sequence coverage of pancreatic peptides. Pancreatic hormones from the flow through analysis of single islets of Langerhans. Detected parts of the sequences are shown in bold.

Precursor	Product	Start	Stop	Sequence
Insulin 1	signal peptide	1	24	MALLVHFLPLLALLALWEPKPTQA
	B-chain	25	54	FVKQHLCGPHLVE**ALYLVCGERGFFYTPKS**
	C-peptide	57	85	**EVEDPQVEQLELGGSPGDLQTLALEVARQ**
	A-chain	88	108	GIVDQCCTSICSLYQLENYCN
Insulin 2	Signal peptide	1	24	MALWMRFLPLLALLFLWESHPTQA
	B-chain	25	54	FVKQHLCGSHLVE**ALYLVCGERGFFYTPMS**
	C-peptide	57	87	**EVEDPQVAQLELGGGPGAGDLQTLALEVAQQ**
	A-chain	90	110	**GIVDQCCTSICSLYQLE**NYCN
Islet amyloid polypeptide	signal peptide	1	19	MMCISKLPAVLLILSVALNHLRA
	propeptide 1	24	35	**TPVRSGSNPQMD**
	peptide	38	74	KCNTATCATQRLANFLVR**SSNNLGPVLPPTNVGSNT**Y
	propeptide 2	75	93	**NAAGDPNRESLDFLLV**
Glucagon	signal peptide	1	20	MKTIYFVAGLLIMLVQGSW**Q**
	glicentin	21	89	**HALQDTEENPRSFPASQTEAHEDPDEMNEDKRHSQGTFTSDYSK**YLDSRRAQDFVQWLMN TKRNRNNIA
	glicentin-related polypeptide	21	50	**HALQDTEENPRSFPASQTEAHEDPDEMNED**
	glucagon	53	81	**HSQGTFTSDYSKYLDSRRAQD**FVQWLMNT
	oxynto-modulin	53	89	**HSQGTFTSDYSKYLDSRRAQD**FVQWLMNTKRNRNNIA
	pro-peptide 1	84	89	NRNNIA
	glucagon-like-peptide 1	92	128	**HDEFERHAEGTFTSDVSSYLEGQAAK**EFIAWLVKGRG
	pro-peptide 2	131	145	DFPEEVAIA**EELGRR**
	Glucagon-like-peptide 2	147	178	**HADGSFSDEMSTILDNLATRDF**INWLIQTKITD
Somato-statin	signal peptide	1	24	MLSCRLQCALAALCIVLALGGVTG
	antrin	25	34	APSDPRLRQF
	neurono-statin	31	43	**LRQFLQKSLAAAT**
	propeptide	35	88	LQK**SLAAATGKQELAK**YFLAELL**SEPNQTENDALEPEDLPQAAEQDEMRLELQR**
	somato-statin 28	89	116	**SANSNPAMAPRE**RKAGCKNFFWKTFTSC
	somato-statin 14	103	116	AGCKNFFWKTFTSC

Peptide YY	signal peptide	1	28	MVAVRRPWPVTVAMLLILLACLGALVDA
	peptide	29	64	**YPAKPEAPGEDASPEELS**RYYASLRHYLNLVTRQRY
	propeptide	69	98	DVPAALFSKL**LFTDDSDSENLPFRPEGLDQW**
Chromo-granin α	signal peptide	1	18	MRS**TAVLALLLCA**GQVFA
	chain	19	463	**LPVNSPMTKGDTK**VMKCVLEVISDSLSKPSPMPVSPECLETLQGDERILSILRHQNLLK**ELQDLALQGAK**ER**AQQPLKQQQPPKQQQQQQQQQQQEQQHSSF**EDELSEV**FENQSPDAKHRDAAAEVPSRDTME**KRK**DSDKGQQDGFEATTEGPRPQAFPEPNQESPMMGDSESPGE**DTATNTQSPTSLPSQEH**VDPQATGDSERGLSAQQQARKAKQEEKEEEEEEEEAVAREKAGPEEVPTAASSSHFHAGYK**AI**QKDDGQSDSQAVDGDGKTEASEALPSEGKGELEHSQQEEDGEEAMVGTPQGLFPQGGKG**R**ELEHKQEEEEEEEERL**SREWEDK**WSRMDQLAKELTAE**K**RLEGEDDPDRSMKLSFRTRAYGFRDPGPQL**RR**GWRPSSREDSVEARSDFEEKKEEEGSANRRAEDQELESL**SAIEAELEKVAHQLQALRRG
	β-granin	19	151	**LPVNSPMTK**GDTKVMKCVLEVISDSLSKPSPMPVSPECLETLQGDERILSILRHQNLLK**ELQDLALQGAK**ER**AQQPLKQQQPPKQQQQQQQQQQQEQQHSSF**EDELSEV**FENQSPDAKHRDAAAEVPSRDTME**
	pancreastin	276	329	**DDGQSDSQAVDGDGKTEASEALPSEGKGELEHSQQEEDGEEAMVGTPQGLFPQG**
	WE-14	358	371	**WSRMDQLAKELTAE**
	catestin	382	402	**RSMKLSFRTRAYGFRDPGPQL**
	GE-25	405	423	**GWRPSSREDSVEARSDFEE**
	serpinin-RRG	435	463	**AEDQELESL**SAIEAELEKVAHQLQALRRG
	serpinin	435	460	**AEDQELESL**SAIEAELEKVAHQLQAL
	p-Glu serpinin precursor	438	460	**QELESL**SAIEAELEKVAHQLQAL
Secreto-granin 1	signal peptide	1	20	MQPAMLLGLLGA**AALAAVSS**
	chain	21	677	**APVDNRDHNEEMVTR**CIIEVL**SNALSKSSVPTI**TPECRQVLKK**SGKEVKGEEKGENQNSKFEV**RLLRDPADASGTR**WASSREDAGAPVEDSQGQTK**VGNEKWTEGGGHSREGVDDQESLRPSNQQASKEAKIYHSEERVGKEREKEEGKIYPMGEHREDAGEEKKHIEDSGEKPNTFSNKRSEASAKKKDES**VARADAHSMELEEKT**HSREQSSQESGEETRRQEKP**QELTDQDQSQEESQEGEEGEEG**EE**GEEGEEDSASEVTK**RRPRHHHGRSGSNKSSYEGHPLSEERRPSPK**ESKEADVATVRLGEKR**SHHLA**HYRASEEEPEYG**EESRSYRGLQYRGRGSEEDRAPRPRSEESQEREYKR**NHPDSELESTANRHGEETEEE**RSYEGANGRQHRGRGRE**PGAHSALDTR**EEKR**LLDEGHYPVRESPIDTA**KRYPQSKWQ**EQEKNYLNYGEEGDQGR**WWQQEEQLGPEESREEVRFPDRQYEPYPITEKRKRLGALFNPYFDPLQWKNSDFEKRGNPDDSFLEDEG**EDRNGVTLTEKNSFPEYNY**DWWER**RPFSEDVNWGYE**KR**SFARAPQLDL**KR**QYDGVAELDQLLHY**RKKADEF**PDFYDSEEQM**

				**GPHQEANDEKARADQR**VLTAEEKKELENLAAMDL<br>ELQKIAEKFSQRG
	PE-11	575	585	**PFSEDVNWGYE**
	CCB peptide	617	677	ADEF**PDFYDSEEQMGPHQEANDEKARADQR**VLT<br>AEEKKELENLAAMDLELQKIAEKFSQRG
Secreto-granin 2	signal peptide	1	20	MAGAKAYRLGAVLLLIHLIFLISGAEAASF
	secreto-granin 2	31	617	QR**NQLLQKEPDLR**LENVQKFPSPEMIRALEYIEKL<br>RQQAHRE**ESSPDYNPYQGV**SVPLQLKENGEESHL<br>AESSR**DALSEDEWMR**IILEALRQAENEPPSAPK**EN**<br>**KPYALNLEK**NFPVDTPDDYETQQWPERKLKHMRF<br>PLM**YEENSRENPF**KRTNEIVEEQYTPQSLATLESV**F**<br>**QELGKLTGPSNQ**KRERVDEEQKLYTDDEDDVYKT<br>NNIAYEDVVGGEDWSPIEEKIETQTQEEVRDSKEN<br>TEKNEQINEEMKR**SGQLGLPDEENRRESK**DQ**LSE**<br>**DASKVIT**YLRRLVNAVGSGRSQSGPNGDRAARLL<br>QKPLDSQSIYQLIEISRNLQIPPEDLIEMLK**AGEKPN**<br>**GLVEPEQDLE**LAV**DLDDIPEADLDRP**DMFQSKMLS<br>KGGYPKAPGRGMVEALPDGLSVEDILNVLGMENV<br>VNQK**SPYFPNQYSQDK**ALMRLPYGPGKSRANQIP<br>K**VAWIPDVESR**QAPYENLNDQELGEYLARMLVKYP<br>ELLNTNQLKRVPSPVS**SEDDLQEEEQ**LEQAIKEHL<br>GPGSSQEMERLAKVSKR**IPVGSLKNEDTPNRQYL**<br>**DEDMLLK**VL**EYLNQEQAEQGREHLA**KRAMENM
	secreto-neurin	184	216	TNEIVEEQYTPQSLATLESVFQ**ELGKLTGPSNQ**
	manserin	527	566	VPSPVSSEDDLQEEEQLEQAIKEHLGPGSSQEMER<br>LAKVS
Secreto-granin 3	signal peptide	1	22	MGFLWTGSWILVLVLNSGPIQA
	chain	23	471	**FPKPEGSQDKSLHN**R**ELSAERPLNEQIAEAEADKI**<br>KKAFPSESKP**SESNYSSVDNLN**LLR**AITEKETVEKE**<br>**RQSI**RSP**PFDNQLNVEDADST**KNRKLIDEYDSTK**S**<br>**GLDHKFQDDPDGLHQDGTPLT**AEDIVHKIATRIYEE<br>NDRGVFDKIVSKLLNLGLITESQAHTLEDEVAEALQ<br>KLISKEANNYEETLDKPTSRTENQDGKIPEKVTP**VA**<br>**AVQDGFTNRENDETVSNTLTLS**NGLER**RTNPHRE**<br>**DDFEELQYFPNF**YALLTSIDSEKEAKEKETLITIMKT<br>LIDF**VKMMVKYGTISPEEGVSYLENLD**ETIALQTKN<br>KLEKNTTDSKSKLFPAPPEK**SQEETDSTKEE**AAKM<br>EKEYGSLKDSTKD**DNSNLGGKTDEATGKT**EAYLE<br>AIRKNIEWLKKHNKKGNKEDYDLSK**MRDFINQQAD**<br>**A**YVEKGILDKEEANAIKRIYSSL
Pro-thymosin α	pro-thymosin α	1	111	MSDAAVDTSSEITTKDLKEK**KEVVEEAENGRDAPA**<br>**NG**NAQN**EENGEQEADNEV**DEEEEEGGEEEEEEE<br>EGDGEEEDGDEDEEAEAPTGK**RVAEDDEDDDVDT**<br>**KKQKTEEDD**
	thymosin α	2	29	SDAAVDTSSEITTKDLKEK**KEVVEEAEN**
Progranulin	signal peptide	1	17	MWVLMSWLAFAAGLVAG
	para-granulin	18	589	TQCPDGQFCPVACCLDQGGANYSCCNPLLDTWP<br>RITSHHLDGSCQTHGHCPAGYSCLLTVSGTSSCCP<br>FSKGVSCGDGYHCCPQGFHCSA**DGKSCFQMSDN**<br>PLGAVQCPGSQFECPDSATCCIMVDGSWGCCPM<br>PQASCCEDRVHCCPHGASCDLVHTRCVSPTGTHT<br>LLKKFPAQKTNRAVSLPFSVVCPDAKTQCPDDSTC

				CELPTGKYGCCPMPNAICCSDHLHCCPQDTVCDLIQSKCLSKNYTTDLLTKLPGYPVKEVKCDMEVSC**PEGYTCCRLNTGAW**GCCPFAKAVCCEDHIHCCPAGFQCHTEKGTCEMGILQVPWMKKVIAPLRLPDPQILKSDTPCDDFTRCPTNNTCCKLNSGDWGCCPIPEAVCCSDNQHCCPQGFTCLAQGYCQKGDTMVAGLEKIPARQTTPLQIGDIGCDQHTSCPVGQTCCPSLKGSWACCQLPHAVCCEDRQHCCPAGYTCNVKARTCEKDVDFIQPPVLLTLGPKVGNVECGEGHFCHDNQTCCKDSAGVWACCPYLKGVCCRDGRHCCPGGFHCSARGTKCLRKKIPRWDMFLRDPVPRPLL
Vaso-pressin-neuro-physin 2-copeptin	signal peptide	1	23	MLARMLNTTLSACFLSLLAFSSA
	Arg-vaso-pressin	24	32	CYFQNCPRG
	neuro-physin	36	128	AISDMELRQCLPCGPGGKGRCFGPSICCADELGCFVGTAEALRCQEENYLPSPCQS**GQKPCGSGGRCAAVGICCSDESCVAEP**ECHDGFFRLT
	copeptin	130	168	AREPSNATQLDGPARALLLRLVQLAGTRESVDSAKPRVY
α-defensin 1	signal peptide	1	29	MKKLVLLFALVLLGFQVQA
	propeptide	20	58	**DSIQNTDEETKTEEQPGEEDQAVSVSFGDPEGTSLQEES**
	peptide	59	93	**LRDLVCYCRSRGCKGRERMNG**TCRKGHLLYTLCCR
α-defensin related sequence 10	signal peptide	1	19	MKKLVLLSAFVLLAFQVQA
	propeptide	20	65	**DSIQNTDEET**KTEEQPGEENQAMSV**SFGDPEGSALQDAAVGMARPC**

Supplementary Table S6. Absorbance data for 96 µFASP-wells loaded with 1 µg of HeLa lysate and quantitated with a Bradford assay.

	A	B	C	D	E	F	G	H
**1**	0,215	0,224	0,213	0,185	0,176	0,221	0,207	0,209
**2**	0,186	0,199	0,18	0,274	0,242	0,231	0,201	0,214
**3**	0,208	0,233	0,202	0,262	0,215	0,252	0,187	0,194
**4**	0,239	0,216	0,247	0,178	0,215	0,242	0,225	0,211
**5**	0,231	0,211	0,179	0,218	0,223	0,255	0,255	0,21
**6**	0,215	0,228	0,222	0,231	0,215	0,2	0,283	0,236
**7**	0,192	0,223	0,181	0,229	0,214	0,166	0,21	0,204
**8**	0,225	0,254	0,219	0,247	0,222	0,229	0,153	0,214
**9**	0,263	0,235	0,193	0,399	0,194	0,173	0,232	0,175
**10**	0,202	0,248	0,203	0,223	0,194	0,218	0,197	0,202
**11**	0,205	0,205	0,201	0,229	0,2	0,221	0,189	0,215
**12**	0,184	0,212	0,231	0,204	0,207	0,209	0,264	0,198
